# The PKD Inhibitor CID755673 Enhances Cardiac Function in Diabetic *db/db* Mice

**DOI:** 10.1371/journal.pone.0120934

**Published:** 2015-03-23

**Authors:** Kylie Venardos, Kirstie A. De Jong, Mansour Elkamie, Timothy Connor, Sean L. McGee

**Affiliations:** 1 Metabolic Remodelling Laboratory, Metabolic Research Unit, School of Medicine, Deakin University, Waurn Ponds, Victoria, Australia; 2 Program for Metabolism and Inflammation, Baker IDI Heart and Diabetes Institute, Melbourne, Victoria, Australia; Stellenbosch University, SOUTH AFRICA

## Abstract

The development of diabetic cardiomyopathy is a key contributor to heart failure and mortality in obesity and type 2 diabetes (T2D). Current therapeutic interventions for T2D have limited impact on the development of diabetic cardiomyopathy. Clearly, new therapies are urgently needed. A potential therapeutic target is protein kinase D (PKD), which is activated by metabolic insults and implicated in the regulation of cardiac metabolism, contractility and hypertrophy. We therefore hypothesised that PKD inhibition would enhance cardiac function in T2D mice. We first validated the obese and T2D *db/db* mouse as a model of early stage diabetic cardiomyopathy, which was characterised by both diastolic and systolic dysfunction, without overt alterations in left ventricular morphology. These functional characteristics were also associated with increased PKD2 phosphorylation in the fed state and a gene expression signature characteristic of PKD activation. Acute administration of the PKD inhibitor CID755673 to normal mice reduced both PKD1 and 2 phosphorylation in a time and dose-dependent manner. Chronic CID755673 administration to T2D *db/db* mice for two weeks reduced expression of the gene expression signature of PKD activation, enhanced indices of both diastolic and systolic left ventricular function and was associated with reduced heart weight. These alterations in cardiac function were independent of changes in glucose homeostasis, insulin action and body composition. These findings suggest that PKD inhibition could be an effective strategy to enhance heart function in obese and diabetic patients and provide an impetus for further mechanistic investigations into the role of PKD in diabetic cardiomyopathy.

## Introduction

Obesity and type 2 diabetes (T2D) are associated with the development of heart failure, which accounts for ~65% of deaths in obese and diabetic patients, based on US statistics [1]. Diabetic cardiomyopathy describes abnormalities in cardiac metabolism that impair contractile function and induce pathological ventricular hypertrophy [2]. The early stages of diabetic cardiomyopathy are characterised by impaired cardiac metabolism, which include insulin resistance, reduced glucose oxidation and increased lipid oxidation [3]. These metabolic alterations result in an energetic deficit that first manifests as diastolic dysfunction, before progressing to systolic dysfunction, and later hypertrophy and heart failure [4]. Existing therapeutics for T2D have limited impact on preventing the development of diabetic cardiomyopathy and some even aggravate the condition [5,6]. Therefore, new therapies that effectively combat the development of diabetic cardiomyopathy are urgently needed.

Protein kinase D (PKD) is activated by metabolic abnormalities, neuroendocrine factors and oxidative stress that are associated with obesity and T2D [7]. It is a serine/threonine kinase with three known isoforms; PKD1–3 [7]. Previously thought to be a Protein kinase C (PKC) isoform termed PKCμ, catalytic domain homology has since distinguished PKD as a member of the calcium calmodulin-dependent kinase (CaMK) family [7]. Activation of PKD involves binding of diacylglycerol to N-terminal cysteine rich domains that relieves autoinhibition of the catalytic domain [8]. Phosphorylation of PKD at a number of sites within the C-terminal catalytic domain confers full PKD activation, culminating in serine 916 autophosphorylation [9]. Numerous growth factors, neuroendocrine factors and oxidative stress are all potent activators of PKD activity [7]. A number of studies have showed that metabolic abnormalities associated with obesity and T2D increase PKD activity. Indeed, PKD activation is increased in cardiomycoytes co-treated with the saturated fatty acid palmitate and high glucose [10]. Similar data is observed in the hearts of male Wistar rats exhibiting hyperglycemia in response to acute (1 day) and chronic (7 day) streptozotocin treatment [10]. In addition, neurohormonal signalling associated with obesity/T2D, such as endothelin-1 and norepinephrine, has also been shown to activate PKD *in vitro* [11]. Changes in PKD activity are also dynamic and regulated in a spatiotemporal manner [11], meaning that quantification of PKD activity in chronic disease states *in vivo* can be challenging. PKD is known to target a number of substrates in cardiomyocytes, including the class IIa histone deacetylases (HDACs) [12] and cardiac troponin I (cTnI) [13], to regulate processes such as metabolism [14], contractility [13] and hypertrophy [12]. Together, these data suggest that PKD could be an effective target for pharmacological modulation in diabetic cardiomyopathy.

A number of small molecule compounds with inhibitory action against PKD have been discovered and synthesised. Of these, the benzoxoloazepinolone family of compounds have high relative potency and specificity against PKD isoforms. The parent benzoxoloazepinolone, termed CID755673, has IC_50_ values of 180, 280 and 227nM against PKD1–3 respectively, and shows ~1000 fold selectivity over closely related PKC kinases [15]. Importantly and unlike many other kinase inhibitors, this compound acts independently of the kinase ATP-binding domain [15], which potentially explains its high degree of specificity. This compound inhibits PKD-regulated processes, including class IIa HDAC phosphorylation [15], and has been used to inhibit prostate cancer growth and motility [16] and pancreatitis *in vivo* [17] in a PKD-dependent manner. The aim of this study was to determine whether the PKD inhibitor CID755673 could prevent cardiac dysfunction in T2D *db/db* mice. Here we report that T2D *db/db* mice are a model of early stage diabetic cardiomyopathy, characterised by both diastolic and systolic dysfunction, without overt alterations in left ventricular morphology, which was associated with elevated PKD2 auto phosphorylation in the fed state and a gene expression signature characteristic of PKD activation. Administration of the PKD inhibitor CID755673 to T2D *db/db* mice for two weeks enhanced indices of both diastolic and systolic left ventricular function and was associated with reduced heart weight. These data suggest that PKD inhibition could be an effective strategy to enhance cardiac function in obese and T2D patients.

## Materials and Methods

### Animal experiments

This study was carried out in strict accordance with animal care guidelines stipulated by the National Health and Medical Research Council (NHMRC) of Australia. The project was approved by the Deakin University Animal Ethics Committee (Application Number G09-2012). All efforts were made throughout the study to minimise animal suffering. Male wild type C57BL6, C57BL6J T2D *db/db* and control heterozygous (*db/-*) mice were obtained from the Animal Resource Centre (Perth, Western Australia) and housed under constant temperature and humidity with 12 h light-dark cycles, with free access to water and food. Untreated *db/-* and *db/db* mice underwent cardiac function assessment at 8 weeks of age and were killed two days later in the fed or fasted state and the heart and right tibia were collected for later analysis. For acute inhibitor studies, C57BL6 mice were administered a single dose of vehicle (5% DMSO in PBS, pH 7.4), or the selective PKD inhibitor CID755673 at 1 or 10mg/kg body weight. Mice were killed one or four hr later and heart collected for later analysis. For chronic inhibitor experiments, 8-week old *db/db* mice received vehicle or CID755673 at 1 or 10mg/kg bodyweight for 16 days, by daily intraperitoneal (i.p.) injection. Body composition by EchoMRI and insulin tolerance was assessed after 12 days of treatment. Insulin tolerance in *db/db* mice was assessed following a 4hr fast by measuring blood glucose obtained from the tail prior to, and 20, 40, 60, 90 and 120 min after insulin (3.5U/kg) administration by i.p. injection. Cardiac function was assessed after 14 days of treatment. At least 2 days later, mice were killed and 4hr fasted blood glucose, the heart and right tibia were collected for later analysis.

### Echocardiography

Echocardiography was performed on mice (n = 7–8/group) under isoflurane inhalation anaesthesia using a Phillips HD15 diagnostic ultrasound system with 15MHz linear-array transducer. Intraventricular septum and left ventricular (LV) posterior wall thicknesses and LV chamber dimensions during both diastole and systole were assessed in M-mode. Doppler imaging provided indices of LV filling, aortic flow and ejection times. Fractional shortening (FS) and ejection fraction, were derived from the M-mode measurements. LV mass was estimated using M-mode data according to the equation by Troy [18], where estimated LV mass = 1.05 ([LVIDD + LVPWD + IVSD]^3^- [LVIDD]^3^), where LVIDD is LV internal diameter at diastole, LVPWD is LV posterior wall thickness at diastole and IVSD is intraventricular septum thickness at diastole.

### Tibia length measurement

Hind legs were covered with 1M NaOH and incubated at 37°C for 5 hours to digest skin, fat and muscle. The tubes were gently agitated every 30 minutes to help aid digestion. After digestion, the kneecap was carefully removed with forceps, and the tibia collected, rinsed, and then dried on paper towel briefly. Tibia length (TL) was then measured using a vernier calliper and used to normalise heart weights.

### Histology

Hearts (n = 3) were rinsed with cold PBS, trimmed and the mid-sections fixed in 10% formalin. Fixed hearts were paraffin embedded, sectioned (4μm thick) then stained with haematoxylin and eosin (H&E). Stained tissue was imaged by light microscopy at 400x magnification and analysed with Zeiss AxioVision software. The size of approximately two hundred cells per LV were measured from 4–6 different fields to assess cardiomyocyte size.

### Western blotting

Heart ventricles (n = 3/group for acute inhibitor studies and n = 7–8/group for all other analyses) were homogenised in ice-cold lysis buffer (50mM Tris pH7.5, 1mM EDTA, 1mM EGTA, 10% glycerol, 1% triton X-100, 50mM NaF, 5mM Na_4_P_2_O_7_, 1mM Na_3_VO_4_, 1mM DTT, protease inhibitor cocktail) and protein content was determined using the BCA method. 30μg of protein was separated by SDS-PAGE and transferred onto PVDF membrane using standard protocols. Blocked membranes were exposed to primary antibodies towards total PKD, pS916 PKD, total cTn1, pS23/24 cTnI, total HDAC5 (Cell Signalling Technology, Danvers, USA), pS498 HDAC [19] and α-tubulin (Sigma-Aldrich, St. Louis, USA). Membranes were visualised using the ChemiDoc XRS+ with Image Lab software. Band intensities of all samples were normalised to an internal control sample that was included on each gel.

### Real time RT-PCR

Heart ventricles (n = 7–8/group) were homogenised in trizol and RNA was isolated using RNeasy columns (Qiagen, Doncaster, Australia). RNA was reverse transcribed using Superscript III chemistry (Life Technologies, Mulgrave, Australia) and cDNA was quantified using Oligreen (Life Technologies, Mulgrave, Australia). Real time RT-PCR was performed using primers specific for Cox7a, PPARα, CPT-1b, PGC-1α, PDK4, GLUT4 and β-actin (sequences published in reference [14]). Gene expression was quantified using the 2Δ^CT^ method and normalisation to β-actin expression, which was not different between groups (S1 and S2 Figs).

### Statistical analyses

All data were analysed using unpaired t-tests or one-way analysis of variance with Tukey’s post-hoc, using GraphPad Prism version 6.0 (Graph-Pad Software Inc., San Diego, USA) and p < 0.05 was considered statistically significant. All data are reported as mean + standard error of the mean (SEM).

## Results

### *db/db* mice displayed impaired cardiac function

**Fig 1:**
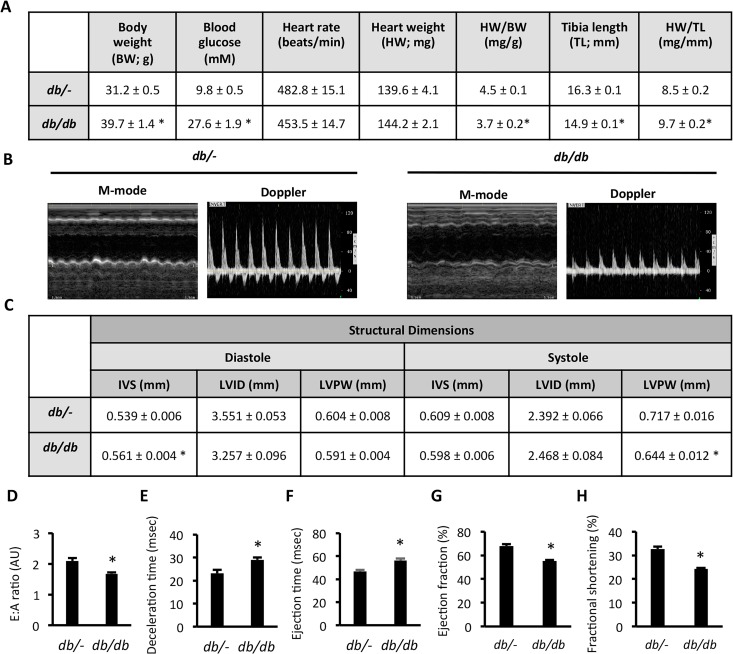
Cardiac dysfunction in *db/db* mice. (A) Physiological and cardiac morphological measures; (B) Representative M-mode and Doppler images; (C) LV Structural dimensions; (D) E:A ratio; (E) Deceleration time; (F) Ejection time; (G) Ejection fraction, and; (H) Fractional shortening in *db/-* and *db/db* mice. Data represented are mean ± SEM, n = 7–8/group. * Denotes significantly different from *db/-* mice (p<0.05). IVS—intraventricular septum thickness; LVID—left ventricular internal diameter; LVPW—left ventricular posterior wall thickness.

The *db/db* mouse model of T2D is a validated model of diabetic cardiomyopathy [20], however the exact phenotype and stage of disease at a given age can be dependent on strain background and other colony specific factors [21]. We therefore evaluated disease progression in 8-week old *db/db* C57BL6J mice through cardiac morphology and function assessment by echocardiography. Compared with control *db/-* mice, *db/db* mice had increased body weight and fasting blood glucose (Fig. 1A), consistent with their T2D phenotype. There was no difference in heart rate, or gross heart weight between *db/-* and *db/db* mice (Fig. 1A). Normalised heart weight to body weight and tibia length were different in *db/db* mice, due to increased body weight and reduced tibia length in these mice (Fig. 1A). Echocardiographic assessment of heart structure and function by M-mode and Doppler imaging (Fig. 1B) showed that LV structural dimensions were similar, except that *db/db* mice had thicker intraventricular septum at diastole and thinner LV posterior wall at systole (Fig. 1C). Diastolic dysfunction was evident in *db/db* mice, characterised by a reduced E:A ratio (Fig. 1D) and increased deceleration time (Fig. 1E). No histological evidence of fibrosis was observed (data not shown). An increased ejection time (Fig. 1F), reduced ejection fraction (Fig. 1G) and decreased fractional shortening (Fig. 1H) also indicated systolic dysfunction in these mice. These data validate the 8-week old *db/db* mouse as a model of developing cardiomyopathy characterised by diastolic and systolic dysfunction, but not pathological hypertrophy.

### *db/db* mice have increased PKD2 autophosphorylation in the fed state and display a gene expression signature consistent with cardiac PKD activation

**Fig 2:**
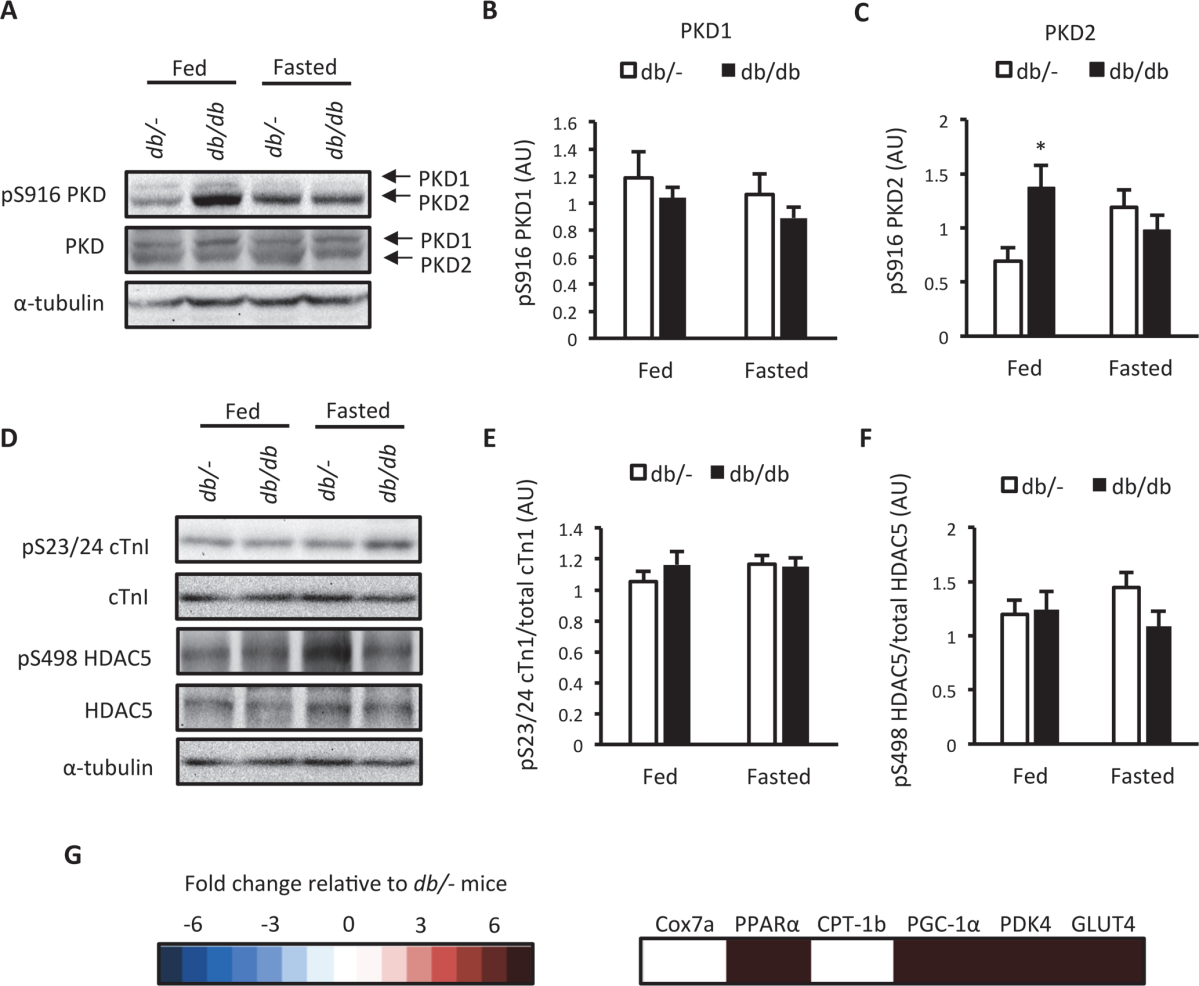
Assessment of PKD activation in *db/db* mice. (A) Representative western blots; (B) S916 PKD1 phosphorylation; (C) S916 PKD2 phosphorylation; (D) Representative western blots; (E) S23/24 cTn1 phosphorylation; (F) S498 HDAC5 phosphorylation in *db/-* and *db/db* mice in the fed or 4 hr fasted state, and; (G) Heat map of a PKD- dependent gene expression signature in 4 hr fasted *db/db* mice. Expression values are relative to *db/-* mice. Data represented are mean ± SEM, n = 7–8/group.

We next assessed ventricular PKD activation in these mice. As PKD activation is sensitive to nutrient availability [10], this was done in mice in the fed and fasted state. No difference was found in autophosphorylation of full length PKD1 at S916 (Fig. 2A and B), which is indicative of PKD activity [22], but we did observe an increase in full length PKD2 autophosphorylation at S916 in fed *db/db* mice (Fig. 2A and C). However, no differences in the phosphorylation of target motifs within the PKD substrates cTn1 and HDAC5 were detected (Fig. 2D, E and F). As PKD activity is regulated in a spatiotemporal and dynamic manner [11], measurement of static PKD autophosphorylation and PKD substrate phosphorylation might not be indicative PKD activation patterns *in vivo*. We therefore examined a gene expression signature indicative of chronic PKD activation in myocytes [14] as a more persistent measure of PKD activation. The expression of 4 out of 6 PKD-dependent genes was elevated in fasted *db/db* mice (Fig. 2G and S1 Fig.). These data suggest that the diastolic and systolic dysfunction observed in *db/db* mice is associated with increased PKD2 activity in the fed state and a gene expression signature consistent with elevated PKD activation.

### The PKD inhibitor CID755673 reduces PKD activation in a time and dose-dependent manner in C57BL6 mice

**Fig 3:**
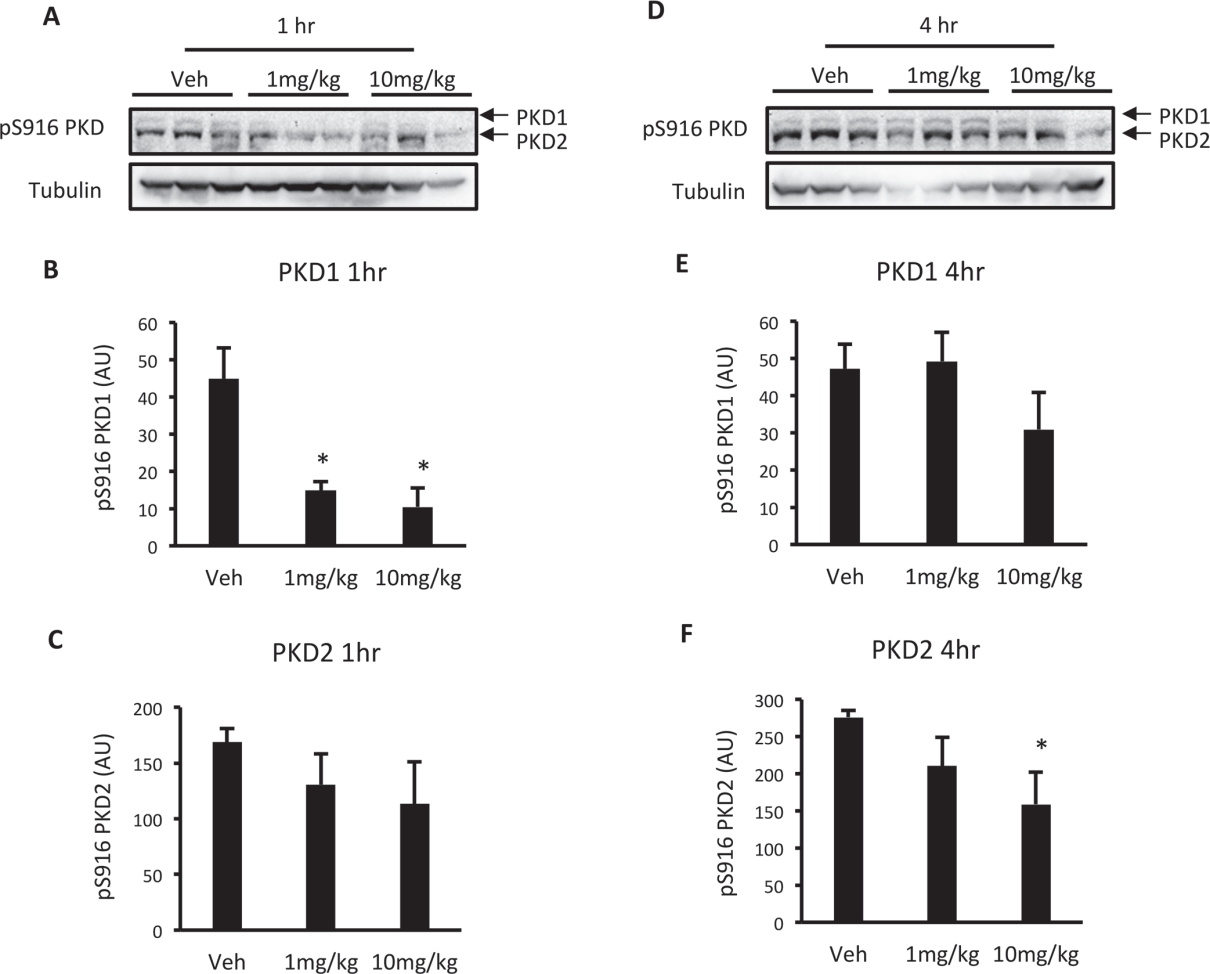
Acute CID755673 administration reduces PKD activity in a time and dose-dependent manner. (A) Representative western blots; (B) S916 PKD1 phosphorylation; (C) S916 PKD2 phosphorylation in the ventricles of wild type C57BL6 mice 1 hr after drug administration. (D) Representative western blots; (E) S916 PKD1 phosphorylation; (F) S916 PKD2 phosphorylation in the ventricles of wild type C57BL6 mice 4 hr after drug administration. Data represented are mean ± SEM, n = 3/group. * Denotes significantly different from vehicle-treated mice (p<0.05).

To determine whether CID755673 reduces PKD activity in the heart, wild type C57BL6 mice were administered vehicle (5% DMSO in PBS) or CID755673 at 1 or 10mg/kg body weight, and were killed 1 and 4 hr after drug administration. One hr after administration, CID755673 reduced PKD1 S916 autophosphorylation at both 1 and 10mg/kg (Fig. 3A and B), while there was no effect on PKD2 (Fig. 3A and C). Four hr after administration, CID755673 had no effect on PKD1 S916 autophosphorylation (Fig. 3D and E), while the 10mg/kg dose reduced PKD2 S916 autophosphorylation (Fig. 3D and F). These data show that .i.p. administered CID755673 reduced PKD activity in a time and dose-dependent manner.

### The PKD inhibitor CID755673 enhanced left ventricular function in *db/db* mice

**Fig 4:**
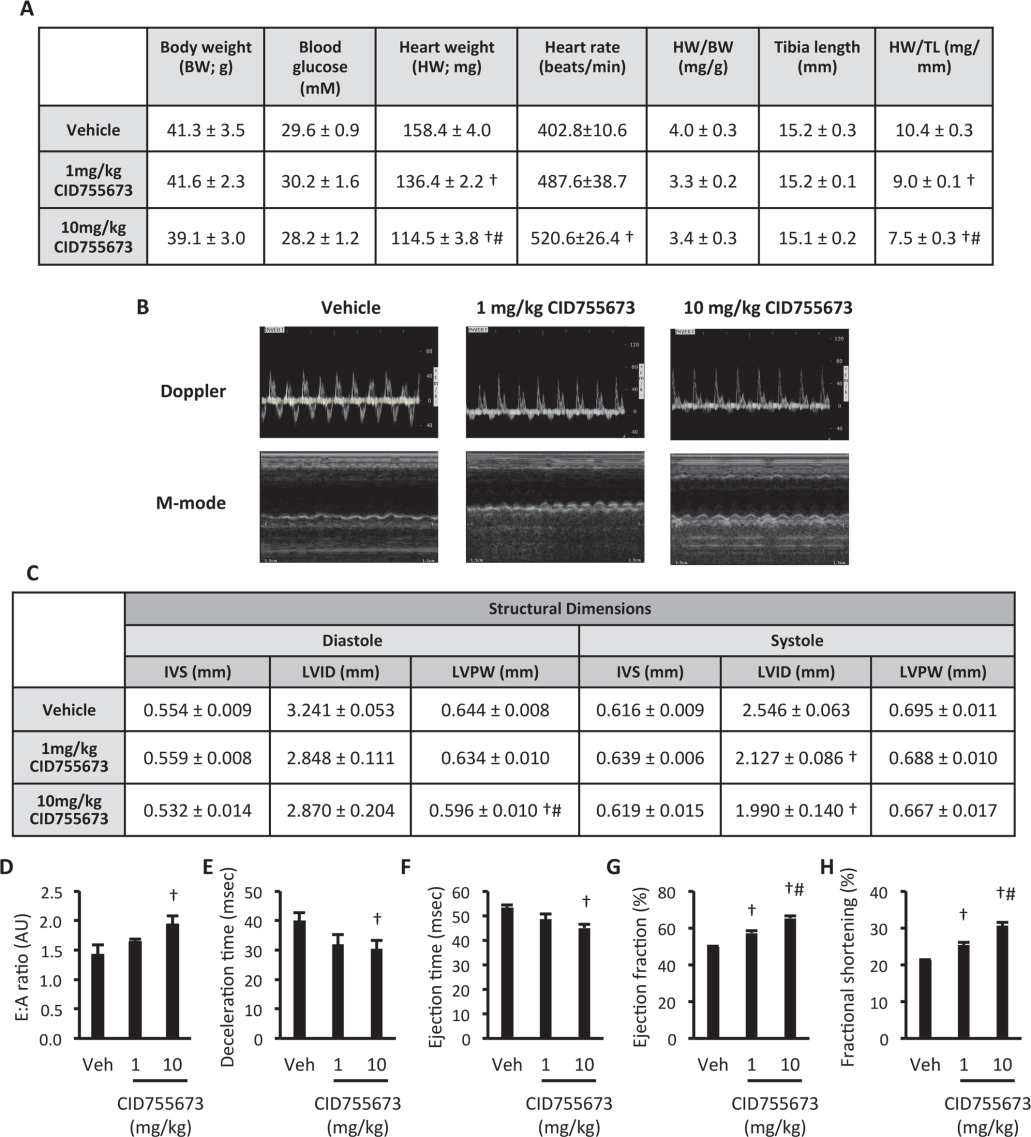
The PKD inhibitor CID755673 enhances cardiac function in *db/db* mice. (A) Physiological and cardiac morphological measures; (B) Representative M-mode and Doppler images; (C) LV Structural dimensions; (D) E:A ratio; (E) Deceleration time; (F) Ejection time; (G) Ejection fraction, and; (H) Fractional shortening in *db/db* mice treated with vehicle, 1mg/kg or 10mg/kg CID755673. Data represented are mean ± SEM, n = 7–8. † Denotes significantly different from vehicle mice (p<0.05). # Denotes significantly different from 1mg/kg CID755673-treated mice. Veh—vehicle; IVS—intraventricular septum thickness; LVID—left ventricular internal diameter; LVPW—left ventricular posterior wall thickness.

*db/db* mice were administered vehicle or CID755673 at 1 or 10mg/kg body weight by daily i.p. injection for two weeks. CID755673 had no effect on body weight or fasting blood glucose, but did induce a dose-dependent decrease in gross heart weight and heart weight when normalised to tibia length (Fig. 4A). CID755673 administered at 10mg/kg increased heart rate when compared with vehicle (Fig. 4A). Echocardiographic assessment of heart structure and function by M-mode and Doppler imaging (Fig. 4B) showed that CID755673 administration at 10mg/kg reduced LV posterior wall (LVPW) thickness at diastole when compared with both vehicle and 1mg/kg CID755673 administration regimens (Fig. 4C). CID755673 administered at both doses reduced LV internal diameter (LVID) at systole. No other differences in LV morphology at either diastole or systole were observed with CID755673 administration. However, CID755673 administration at 10mg/kg enhanced both diastolic function, signified by an increased E:A ratio (Fig. 4D) and reduced deceleration time (Fig. 4E), and systolic function, demonstrated by decreased ejection time (Fig. 4F). Dose-dependent increases in indices of systolic function, such as ejection fraction (Fig. 4G) and fractional shortening (Fig. 4H) were also observed. Together, these data show that the PKD inhibitor CID755673 enhanced both diastolic and systolic cardiac function in *db/db* mice, which was associated with a reduction in gross heart weight and reduced LVID at systole.

### CID755673 administration was associated with an altered PKD-dependent gene expression signature, consistent with PKD inhibition

**Fig 5:**
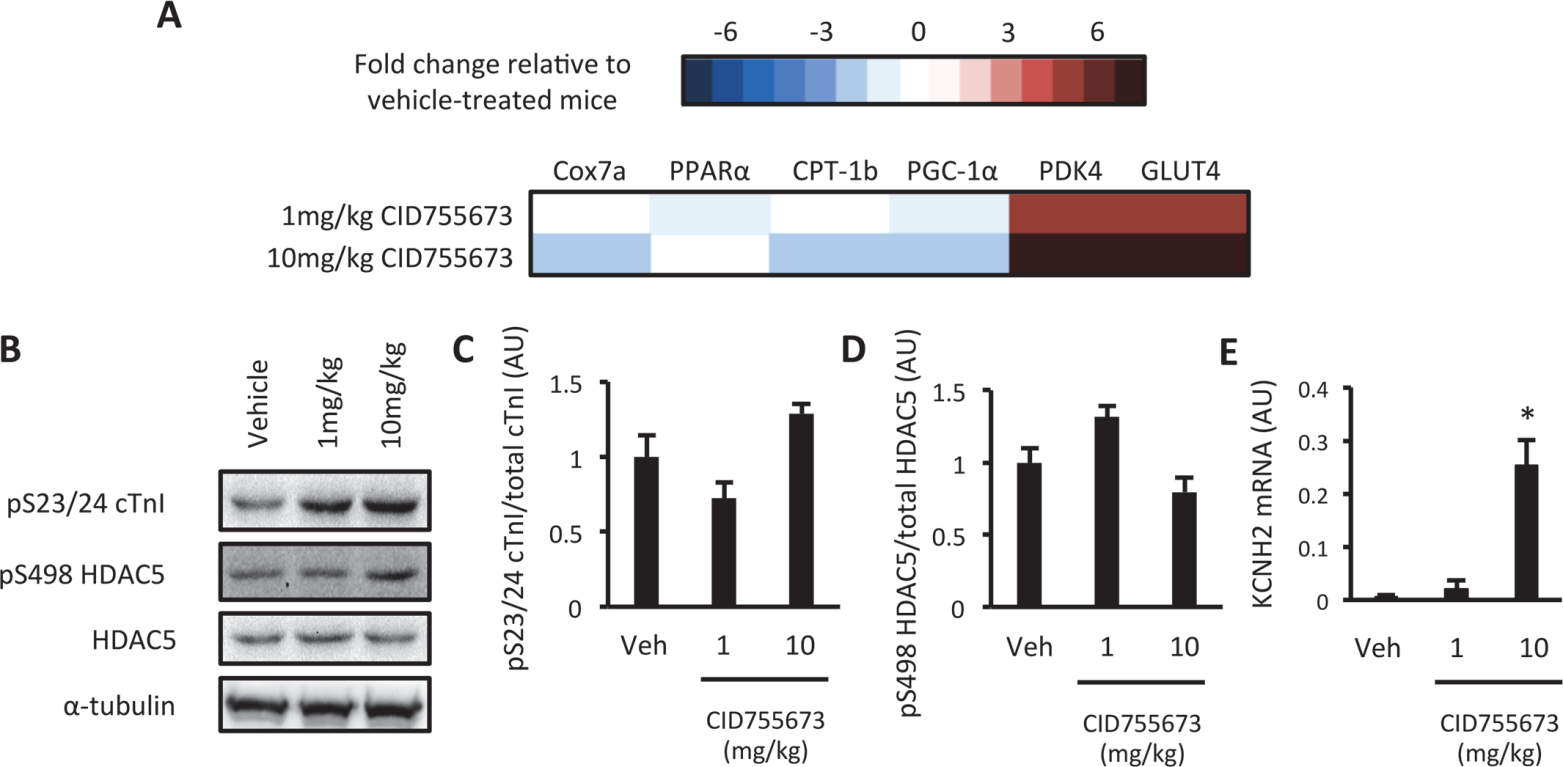
Assessment of PKD activation in CID755673-treated *db/db* mice. (A) Heat map of a PKD- dependent gene expression signature; (B) Representative western blots; (C) S23/24 cTn1 phosphorylation; (D) S498 HDAC5 phosphorylation; (E) KCNH2 gene expression in vehicle, 1mg/kg and 10mg/kg CID755673-treated *db/db* mice. Expression values are relative to *db/-* mice. Data represented are mean ± SEM, n = 7–8/group. * Denotes significantly different from vehicle-treated mice (p<0.05).

We next assessed PKD-dependent signalling in the ventricles of vehicle and CID755673-treated mice that had undergone a 4hr fast (Fig. 4A). We first assessed whether CID755673 administration altered our PKD-dependent gene expression signature. The expression of 4 PKD-dependent genes was reduced by CID755673 administration across both doses, suggestive of PKD inhibition, while the remaining 2 genes were markedly increased (Fig. 5A and S2 Fig.). No differences in S23/S24 cTn1 (Fig. 5B and C) and S498 HDAC5 (Fig. 5B and D) phosphorylation was observed with CID755673 administration. However, we did observe a significant increase in the expression of the KCNH2 gene (Fig. 5E), which encodes a potassium voltage-gated channel involved in cardiomyocytes electrical function that is thought to be suppressed by PKD in obesity [23]. Together, these data suggest some impairment in downstream PKD signalling in CID755673 treated *db/db* mice.

### CID755673-mediated reduction in heart size was not specific for the LV

**Fig 6:**
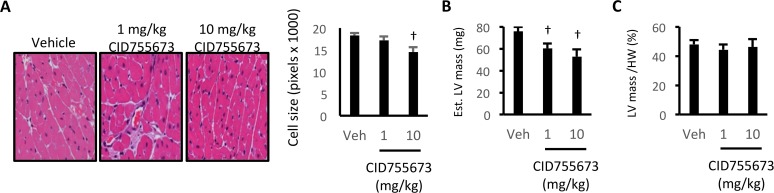
The CID755673-mediated reduction in heart size is not specific for the LV. (A) Representative cell size images and quantification; (B) Estimated gross LV mass, and; (C) LV mass normalised to heart weight in *db/db* mice treated with vehicle, 1mg/kg or 10mg/kg CID755673. Data represented are mean ± SEM, n = 3–8/group. † Denotes significantly different from vehicle mice (p<0.05).

We further assessed the cardiac remodelling that contributed to the reduced heart size and enhanced cardiac function observed following CID755673 administration. To examine the specific effects on heart morphology, we examined LV cell size by histology (Fig. 6A). CID755673 administration at 10mg/kg reduced LV cell size (Fig. 6A), and calculated LV mass from M-mode echocardiography was reduced at both drug doses (Fig. 6B). However calculated LV mass was not different when normalised to total heart weight (Fig. 6C), suggesting that the reduction in heart weight was consistent across all chambers of the heart. These alterations could not be ascribed to changes in body composition or insulin tolerance (S3 Fig.), suggesting that gross alterations in systemic metabolism were not involved. Together, these data show that the PKD inhibitor CID755673 enhanced cardiac systolic and diastolic function in diabetic cardiomyopathy in association with reduced heart size, but with only minor alterations in LV internal structural dimensions.

## Discussion

Cardiovascular disease and heart failure are major comorbidities for T2D, accounting for ~65% of deaths due to T2D [1]. Clearly new therapeutic strategies are needed to attenuate the incidence of cardiovascular disease mortality in obese and T2D patients, which would be most effectively targeted in the early stages of diabetic cardiomyopathy development. Here we showed that the obese and T2D *db/db* mouse on a C57BL6J background at 8 weeks of age represents a model of developing diabetic cardiomyopathy characterised by systolic and diastolic dysfunction, in the absence of compensatory pathological hypertrophy. Therefore, this mouse model is useful for the study of developing diabetic cardiomyopathy and for experimental drug studies aimed at preventing the disease in its early stages. We observed increased PKD2 activation in the fed state in these *db/db* mice and components of a gene expression signature of PKD activation were elevated. Administration of the PKD inhibitor CID755673 to *db/db* mice for 2-weeks improved both systolic and diastolic cardiac function, and was associated with a reduction in gross heart size. Together, these data suggest that compounds with inhibitory activity towards PKD could be effective in enhancing cardiac function in diabetic cardiomyopathy, when administered in the early stages of the disease.

The finding that PKD2 autophosphorylation was increased in *db/db* mice in the fed state provides further evidence that PKD isoforms are sensitive to alterations in substrate availability. The finding that PKD2 activation was not different between control and *db/db* mice after 4 hr of fasting is consistent with other observations showing that PKD activation is dynamic [24] and can be regulated in a spatiotemporal manner [11]. It is possible that static measures of PKD activity in whole ventricle lysates are not spatiotemporally sensitive enough to accurately assess PKD activity *in vivo*. Therefore, we assessed a gene expression signature of PKD activation, which we previously identified in myocytes following constitutively active PKD expression [14], as a more stable measure of PKD activation in these hearts. Although consisting primarily of metabolic genes, we observed that 4 of 6 genes of the expression signature were elevated in diabetic *db/db* mice when compared with control mice. It should be noted that the 2 remaining genes, PDK4 and GLUT4, were substantially increased in response to CID755673. However, it is possible that these genes were up-regulated by secondary mechanisms related to alterations in glucose flux following the cardiac remodelling by CID755673. Clearly further research will be required to more clearly define PKD activation patterns in the development of diabetic cardiomyopathy *in vivo*, including any time course of activation and the putative factors contributing to any increases in PKD activity. An intriguing finding from this study was that despite measures of PKD activation and the profound effects of CID755673 on cardiac function in *db/db* mice, we did not observe any alterations in the phosphorylation of two putative downstream PKD targets in these same mice. cTn1 and HDAC5 are both phosphorylated by PKD and are thought to regulate contractility and hypertrophy and metabolism responses, respectively. However, the effect of CID755673 on the cardiomyopathy observed in *db/db* mice could be due to the regulation of other, including as yet unidentified, PKD targets.

The underlying mechanisms by which CID755673 enhanced both diastolic and systolic function are unknown. However, in the context of developing diabetic cardiomyopathy, PKD is known to regulate both cardiac metabolism and contractility. Indeed, loss of PKD1 through either siRNA or through genetic knockout is associated with a loss of GLUT4 translocation and glucose uptake [25]. This is consistent with our own data in myocytes showing that constitutive PKD activation enhances glucose oxidation [14]. However, as the development of diabetic cardiomyopathy is associated with a shift from glucose to lipid metabolism, this would suggest that PKD inhibition would be unlikely to result in restoration of glucose metabolism that in turn prevents or reverses the diabetic cardiomyopathy phenotype. However, PKD has also been linked to increased fatty acid uptake under insulin resistant conditions [26], suggesting that impairment in PKD activity could contribute to cardiac lipotoxicity in insulin resistance. An alternative mechanism by which CID755673 had its effects on diastolic and systolic function could be through modulation of cardiac contractility. Indeed, heart rate was increased in CID755673-treated animals, possibly indicating that contractile processes and/or calcium handling were improved. While we did not see any difference in cTn1 phosphorylation in any of our analyses, we did observe an increase in the expression of the KCNH2 gene, which encodes the Kv11.1 voltage-gated potassium channel that is required for normal cardiac electrical conduction. PKD has been found to suppress this gene in obesity [23] and could be one mechanism by which PKD inhibition enhanced both diastolic and systolic function. It should also be noted that PKD inhibition can be detrimental in settings of ischemia-reperfusion injury [27], which suggests that the protective effects of CID755673 in *db/db* mice are likely to be specific to the disease itself.

Treatment of *db/db* mice with CID755673 resulted in a dose dependent reduction in gross heart size. Although this was associated with a reduction in LV cell size and estimated LV mass, the reduction in mass was not specific for the LV as there was no change in LV mass when normalised to heart weight. This is consistent with our initial characterisation of the *db/db* model that showed no pathological hypertrophy and suggests that a fundamental process controlling cardiomyocyte size was affected by CID755673 treatment. Indeed, a recent report suggests that PKD3 can directly phosphorylate and activate S6K1, in an mTOR-independent manner, which contributes to breast cancer cell growth [28]. Whether this mechanism explains the whole heart phenotype observed in the present study is unclear, however it should be noted that the reduction in heart size was not associated with deleterious effects on heart function but instead improvements in cardiac function. This could suggest that the reduction in heart weight in CID755673-treated mice was related to decompensation due to enhanced function. Indeed, the reduction in heart size and enhancement in cardiac function is remarkably similar to that observed with loss of phospholamban in disease states [29].

In conclusion, here we showed that the 8 week old *db/db* mouse is a model of developing diabetic cardiomyopathy characterised by diastolic and systolic dysfunction, but not pathological hypertrophy, and PKD2 activation in the fed state. Diastolic and systolic function were enhanced following 2-weeks of treatment with the CID755673 PKD inhibitor and was associated with a reduction in gross heart weight. Current first line treatments for T2D are typically focussed on controlling blood glucose levels, and have varying effects on diabetic cardiomyopathy [5,30,31]. The remaining high prevalence of heart failure in obesity and T2D suggests that additional therapeutic interventions are required. The proof-of-principle findings in the present study suggest that small molecules that have inhibitory activity towards PKD could be promising therapeutics to reduce heart disease in obese and diabetic patients. In addition to genetic gain and loss of function studies to determine the exact role of PKD in diabetic cardiomyopathy, further toxicity and efficacy testing of other PKD inhibitors and long term drug studies are warranted and could support a role for PKD inhibition as a therapeutic treatment option for developing diabetic cardiomyopathy.

## Supporting Information

S1 Fig PKD-dependent gene expression in *db/-* and *db/db* mice. (A) Cytochrome C oxidase subunit 7a (Cox7a); (B) Peroxisome proliferator-activated receptor alpha (PPARα); (C) carnitine palmitoyltransferase 1b (CPT-1b); (D) PPAR gamma coactivator 1 alpha (PGC-1α); (E) pyruvate dehydrogenase kinase isoform 4 (PDK4); (F) facilitative glucose transporter isoform 4 (GLUT4), and; (G) β-actin gene expression in control *db/-* and type 2 diabetic *db/db* mice. Gene expression levels in A-F were normalised to β-actin expression. Data are represented as means ± SEM. *Denotes significantly different from *db/-* mice (p<0.05).
(TIF)

S2 Fig PKD-dependent gene expression in *db/db* mice treated with vehicle, 1mg/kg or 10 mg/kg CID755673. (A) Cytochrome C oxidase subunit 7a (Cox7a); (B) Peroxisome proliferator-activated receptor alpha (PPARα); (C) carnitine palmitoyltransferase 1b (CPT-1b); (D) PPAR gamma coactivator 1 alpha (PGC-1α); (E) pyruvate dehydrogenase kinase isoform 4 (PDK4); (F) facilitative glucose transporter isoform 4 (GLUT4), and; (G) β-actin gene expression in vehicle (Veh), 1mg/kg CID755673 and 10mg/kg CID755673 treated *db/db* mice. Gene expression levels in A-F were normalised to β-actin expression. Data are represented as means ± SEM. *Denotes significantly different from vehicle treated mice (p<0.05).
(TIF)

S3 Fig Metabolic and body composition measures in *db/db* mice treated with vehicle, 1 mg/kg or 10 mg/kg CID755673. (A) Change (Δ) in blood glucose (mM) during an insulin tolerance test in vehicle, 1mg/kg CID755673 and 10mg/kg CID755673 treated *db/db* mice. Mice were administered 3.5U/kg insulin via intraperitoneal injection. (B) Body composition in vehicle, 1mg/kg CID755673 and 10mg/kg CID755673 treated *db/db* mice, assessed by EchoMRI. Data are represented as mean ± SEM.
(TIF)
