# Supplementary figures and images for: The PKD Inhibitor CID755673 Enhances Cardiac Function in Diabetic db/db Mice
Source: PLoS One. 2015 Mar 23;10(3):e0120934. doi: 10.1371/journal.pone.0120934 (PMC4370864; doi:10.1371/journal.pone.0120934)

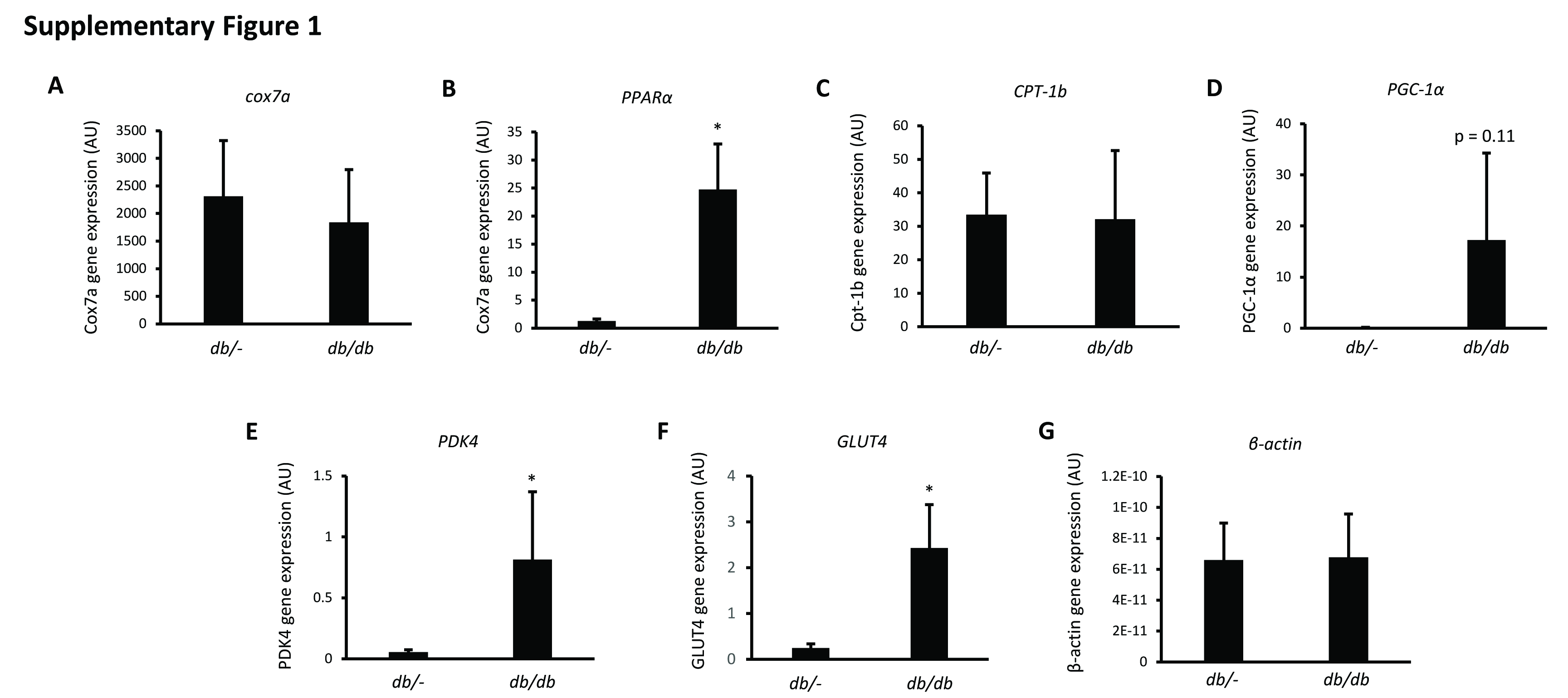

Supplement: S1 Fig — (A) Cytochrome C oxidase subunit 7a (Cox7a); (B) Peroxisome proliferator-activated receptor alpha (PPARα); (C) carnitine palmitoyltransferase 1b (CPT-1b); (D) PPAR gamma coactivator 1 alpha (PGC-1α); (E) pyruvate dehydrogenase kinase isoform 4 (PDK4); (F) facilitative glucose transporter isoform 4 (GLUT4), and; (G) β-actin gene expression in control db/- and type 2 diabetic db/db mice. Gene expression levels in A-F were normalised to β-actin expression. Data are represented as means ± SEM. *Denotes significantly different from db/- mice (p<0.05). (TIF) [file pone.0120934.s001.tif]

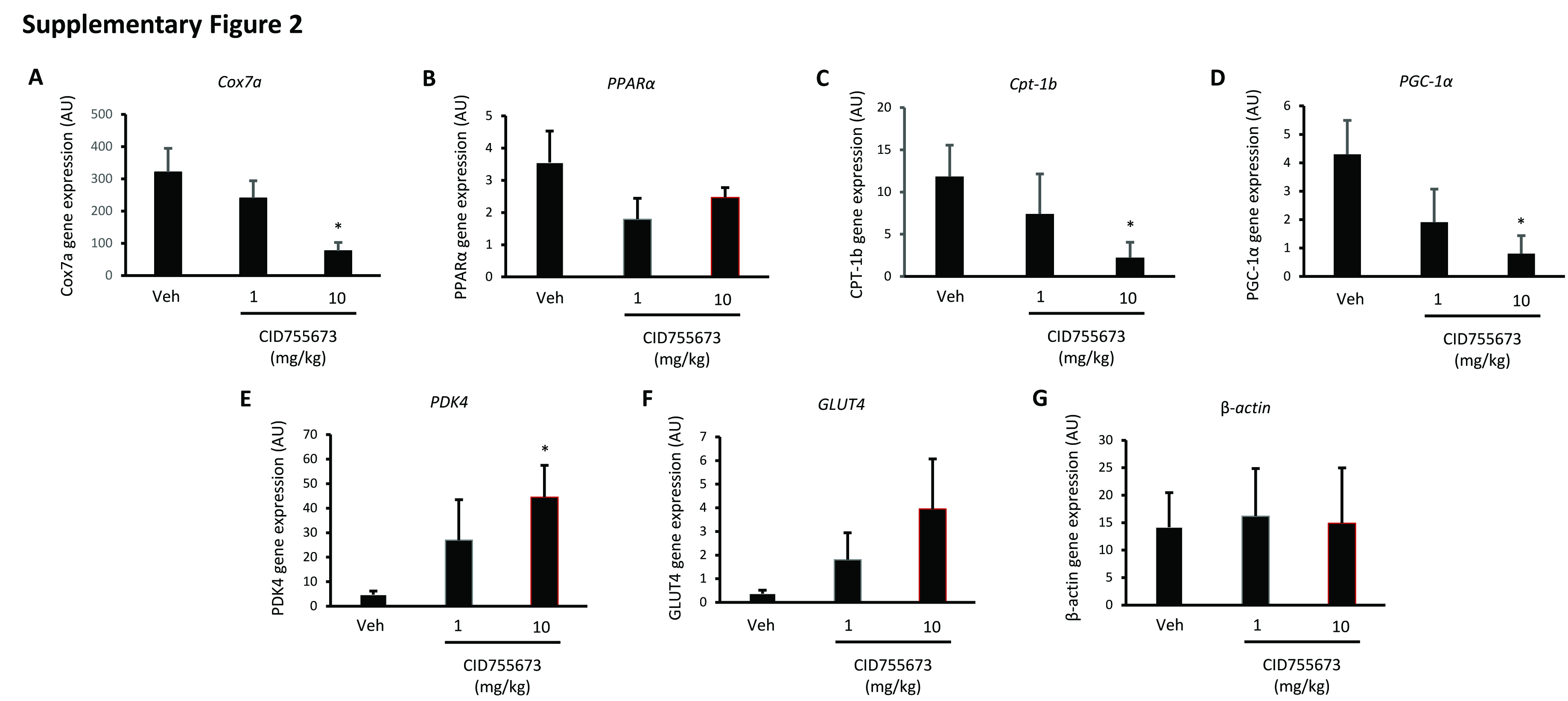

Supplement: S2 Fig — (A) Cytochrome C oxidase subunit 7a (Cox7a); (B) Peroxisome proliferator-activated receptor alpha (PPARα); (C) carnitine palmitoyltransferase 1b (CPT-1b); (D) PPAR gamma coactivator 1 alpha (PGC-1α); (E) pyruvate dehydrogenase kinase isoform 4 (PDK4); (F) facilitative glucose transporter isoform 4 (GLUT4), and; (G) β-actin gene expression in vehicle (Veh), 1mg/kg CID755673 and 10mg/kg CID755673 treated db/db mice. Gene expression levels in A-F were normalised to β-actin expression. Data are represented as means ± SEM. *Denotes significantly different from vehicle treated mice (p<0.05). (TIF) [file pone.0120934.s002.tif]

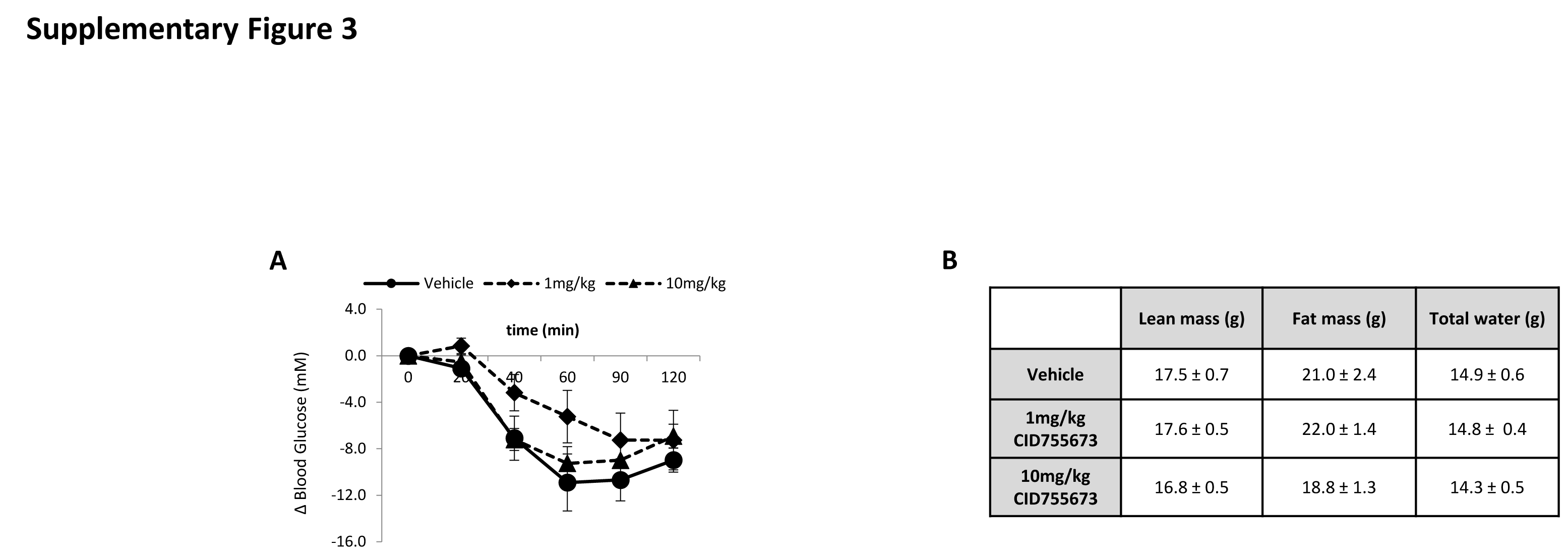

Supplement: S3 Fig — (A) Change (Δ) in blood glucose (mM) during an insulin tolerance test in vehicle, 1mg/kg CID755673 and 10mg/kg CID755673 treated db/db mice. Mice were administered 3.5U/kg insulin via intraperitoneal injection. (B) Body composition in vehicle, 1mg/kg CID755673 and 10mg/kg CID755673 treated db/db mice, assessed by EchoMRI. Data are represented as mean ± SEM. (TIF) [file pone.0120934.s003.tif]
